# Parenting Behaviors as Mediators of the Association Between Parental Internalizing Symptoms and Child Externalizing Symptoms

**DOI:** 10.1007/s10578-022-01462-0

**Published:** 2022-10-28

**Authors:** Marie-Theres Klemp, Christina Dose, Christopher Hautmann, Lea T. Jendreizik, Judith Mühlenmeister, Julia Plück, Laura Wähnke, Manfred Döpfner

**Affiliations:** 1 Faculty of Medicine and University Hospital Cologne, School for Child and Adolescent Cognitive Behavior Therapy (AKiP), University of Cologne, Pohligstr. 9, 50969 Cologne, Germany; 2 Faculty of Medicine and University Hospital Cologne, Department of Child and Adolescent Psychiatry, Psychosomatics and Psychotherapy, University of Cologne, Robert-Koch-Str. 10, 50931 Cologne, Germany

**Keywords:** Attention-deficit/hyperactivity disorder (ADHD), Oppositional defiant disorder (ODD), Parenting behavior, Mediation analysis

## Abstract

This study analyzes whether the association between parental internalizing symptoms (depression, anxiety, stress) and child symptoms of attention-deficit/hyperactivity disorder (ADHD) or oppositional defiant disorder (ODD) is mediated by positive and negative parenting behaviors. Cross-sectional data of 420 parents of children (age 6–12 years) with elevated levels of externalizing symptoms were collected in a randomized controlled trial. Measures included parent ratings of their internalizing symptoms and parenting behaviors and of their child’s externalizing symptoms. Two mediation models were examined, one including ADHD symptoms and one including ODD symptoms as the dependent variable. Parental internalizing symptoms were modeled as the independent variable and positive and negative parenting behaviors were modeled as parallel mediators. Regression analyses support negative parenting behavior as a mediator of the association between parental internalizing symptoms and child ODD symptoms. For the ADHD model, no significant mediator could be found. Future studies should use prospective designs and consider reciprocal associations.

**Supplementary Information:**

The online version contains supplementary material available at 10.1007/s10578-022-01462-0.

## Introduction

Externalizing disorders such as attention-deficit/hyperactivity disorder (ADHD) and oppositional defiant disorder (ODD) are common mental disorders in childhood, with worldwide pooled prevalence rates of 3.4% for ADHD and 3.6% for ODD among children and adolescents [1]. ADHD is characterized by developmentally inappropriate levels of hyperactivity, impulsivity, and/or inattention [2, 3], while ODD is defined as a pattern of short-tempered mood and anger, irritability, and confrontational behavior, but without severe violent or harmful behavior [2, 3]. The two disorders show a high degree of comorbidity with each other [4–6].

A number of studies have demonstrated associations between parental internalizing symptoms and child externalizing behavior problems. For instance, meta-analyses revealed higher rates of both internalizing and externalizing symptoms in parents of children with versus without ADHD [7, 8], with the highest rates found for parental ADHD, depression, and anxiety symptoms [7]. Moreover, a recent study using longitudinal data reported that increasing parental mental health problems were related to increasing ADHD symptoms of the child over time [9, 10]. Particularly considering internalizing symptoms of the parents, previous research found higher levels of anxiety, depression, and stress in parents of children screening positive for ADHD compared to screening-negative controls [11]. Additionally, several longitudinal studies have reported associations between maternal depressive symptoms and later child externalizing behavior problems [6, 12–14], and maternal depressive symptoms were identified as a risk factor for high trajectories of hyperactivity-impulsivity and inattention symptoms in the child [15]. While some studies support the presence of a unidirectional influence from parental depressive symptoms to child externalizing symptoms [13, 16], others hint at the reverse effect, insofar as child externalizing behavior, such as the tendency for tantrums and emotional dysregulation, affects parental mood [17, 18]. Indeed, mothers of children with externalizing behavior problems such as ADHD were found to show high levels of parenting stress over time, and child behavior problems and parental depressive symptoms both predicted parental stress [9]. However, other longitudinal studies have demonstrated that child and parental psychopathologies influence one another, thus suggesting that the effect is reciprocal in nature [19–21].

To improve the prevention and treatment of child externalizing behavior disorders, it would be useful to know the mechanisms that account for the association between parental internalizing symptoms and child externalizing symptoms. It is most likely that the association between parental and child symptoms is the result of a complex interplay of different factors. In an integrative, developmentally sensitive model, Goodman and Gotlib [22] suggested four mechanisms through which maternal depressive symptoms exert an effect on child psychopathology: (1) heritability, (2) innate dysfunctional neuroregulatory mechanisms, (3) exposure to the mother’s negative and/or maladaptive cognitions, behaviors, and affect, and (4) exposure to a stressful environment. This model can probably be generalized to other maternal mental health conditions [22, 23]. In terms of the development or modification of psychosocial interventions for the treatment of child psychopathology, the mechanism relating to the mother’s dysfunctional cognitions, behavior, and affect is of particular interest, as interventions may be designed to target these constructs. One hypothesis relating to this mechanism is that parental symptoms lead to specific parenting behaviors, which in turn affect the symptoms of the child. To test such a hypothesis, mediation analyses can be used [24, 25].

On a theoretical level, the association between parenting practices and child externalizing symptoms might be explained using the coercive family process model by Patterson (1983, 1989), which models the mutual reinforcement of dysfunctional parenting practices and disruptive behaviors of the child, and thus explains the development and escalation of these behaviors [26]. In line with this model, numerous studies have revealed associations between parenting practices and ODD symptoms or externalizing symptoms in general. For instance, parental rejection and overprotection as perceived by the child were shown to precede externalizing behavior problems in general [27, 28], and overreactive parenting practices were found to be prospectively related to the presence of ODD symptoms [14]. Moreover, harsh parenting is very likely to lead to antisocial behavior in children [29].

Furthermore, parents of children with externalizing disorders were found to show a more inconsistent and hostile parenting style compared to a control group [11]. Another study reported that in children diagnosed with ADHD, both comorbid ODD and conduct disorder were significantly associated with maternal negative/ineffective discipline [30]. Although the model by Patterson was not designed to explain the development of ADHD symptoms, some aspects of negative parenting behavior have also been linked to this disorder. For instance, inconsistent discipline [31], overreactive parenting [14, 32] and rejecting parenting [33] have been found to be predictive of later ADHD symptoms.

On the other hand, aspects of positive parenting practices seem to act as protective factors regarding the development of different types of externalizing behavior. For example, positive parenting practices have been associated with fewer future conduct problems [6] and were found to have a positive impact on ADHD symptoms [34]. In a longitudinal study, warm parenting by adoptive mothers predicted lower levels of later child externalizing problems [35], and a study of clinic-referred families reported an association between higher parental involvement and lower levels of later hyperactivity and inattention [32]. Moreover, cross-sectional data revealed that parents of children screening negative for ADHD demonstrated more warmth than parents of children with positive screening results [11]. Parental symptoms of depression, anxiety, and stress may contribute to dysfunctional parenting practices. For instance, depressive mothers tend to report fewer firm and consistent parenting behaviors, less warm and nurturing parenting, and fewer positive parenting practices than do non-depressed mothers [36]. Moreover, mothers experiencing depressive symptoms often show a decline in positive parenting practices [37], and mothers with recurrent episodes of depression reported more anger and hostility and less tolerance towards their toddlers [38]. Similarly, maternal anxiety was found to lead to less parental warmth and less positive engagement [39].These parenting practices might permit interactions within the family which reinforce disruptive behaviors of the child, as emphasized in the model by Patterson.

To the best of our knowledge, only a small number of studies have directly examined parenting practices as potential mediators of the association between parental symptoms of depression, anxiety, and stress and child externalizing symptoms, with some studies detecting mediating effects and others reporting no such effects. Analyzing a community sample, Trepat et al. [40] found that corporal punishment mediated the (non-significant) association between maternal anxiety-depression symptoms and child ODD symptoms in preschool-age girls but not boys. The relation between paternal internalizing symptoms and child ODD was not mediated by paternal parenting practices [40]. In a large community sample, Elgar et al. [41] demonstrated that the effect of self-rated parental depression on later self-rated child externalizing symptoms was mediated by child-rated parental nurturance and rejection. Using longitudinal data of a community sample of school-age children, Dette-Hagenmeyer and Reichle [42] reported that mothers’ inconsistent use of discipline mediated the longitudinal association between the mothers’ depressive symptoms and the children’s ODD and hyperactivity symptoms [42]. When considering fathers, the authors found that inconsistent discipline (positively) mediated the association between paternal depressive symptoms and child ODD symptoms, and positive parenting behavior (negatively) mediated the association between paternal depressive symptoms and child hyperactivity [42]. To our knowledge, only one study has examined the mediation of the association between parental internalizing symptoms and child externalizing behavior in a clinical sample [43]. Based on cross-sectional data from a sample of mother–child dyads (child age 8–12 years) referred for treatment, Van Doorn et al. [43] demonstrated a strong association between self-reported maternal depressive symptoms and maternal reports of children’s internalizing and externalizing mental health problems. However, different aspects of observed mother–child interactions (i.e., maternal warmth and maternal psychological control) did not mediate the relation between maternal depressive symptoms and child mental health problems. The findings were limited by the sample size (*n* = 111) and the authors suggested that the study may have been underpowered [43]. Moreover, the results were further limited by the low internal consistency of the scale used to assess parental warmth.

The present study examined the mediation of the impact of a general measure of parental symptoms of depression, anxiety, and stress on child ADHD and ODD by positive parenting behaviors (e.g., the use of praise, encouragement, joint play, supportive strategies; cf. [44]) and negative parenting behaviors (e.g., verbal criticism, harshness; cf. [44]). In contrast to previous studies, we considered a large clinical sample of children with elevated levels of externalizing behavior problems. Moreover, while most previous analyses concentrated on the effects of parental depressive symptoms, we expanded the previously tested models by also including parental symptoms of anxiety and stress, thus considering parental internalizing symptoms on a more general level.

In particular, we hypothesized that more severe symptoms of parental depression, anxiety, and stress would predict a lower level of positive parenting behaviors and a higher level of negative parenting behaviors, which would in turn lead to more severe ADHD or ODD symptoms, respectively, in the child. ADHD symptoms seem to be more strongly determined by genetic influences than do ODD symptoms [45, 46], whereas a recent twin study demonstrated a strong influence of environmental factors on the development of ODD symptoms [30, 47]. Moreover, the aforementioned coercive family process model was originally conceived to explain the development of disruptive symptoms (and not the development of ADHD core symptoms). Thus, the association between parenting practices and child symptoms seems to be more strongly pronounced in children with ODD than in children with ADHD [48–50].

Accordingly, we expected the effects of parental symptoms on child ODD symptoms to be more strongly mediated by parenting behaviors compared to the respective effects on child ADHD symptoms. Moreover, from an exploratory perspective, we examined the relative contribution of positive and negative parenting practices to this mediation process.

## Methods

### Study Design and Participants

The data for the current analyses were gathered as part of a randomized controlled trial [RCT] on the efficacy of a web-assisted self-help program [WASH] for parents of children with symptoms of ADHD and/or ODD [51]. The RCT was registered at the German Clinical Trials Register (identifier: DRKS00013456) and approved by the Ethics Committee of the University Hospital Cologne, Germany. We compared three study conditions: treatment as usual, WASH plus treatment as usual, and WASH plus telephone-based support. The analyses presented in this article used baseline data from participants in all three study conditions.

For recruitment purposes, study information was sent to 5015 pediatricians and child and adolescent psychiatrists in Germany, who could then register eligible participants. Recruitment took place between December 2017 and February 2020. To participate in the RCT, families had to meet the following inclusion criteria: (a) the child was aged between 6 and 12 years, (b) the referring health care provider had diagnosed the child with an externalizing behavior disorder or suspected the diagnosis of an externalizing disorder, and (c) the child demonstrated an elevated level of externalizing symptoms. Externalizing symptoms were assessed by a clinician using the semi-structured Clinical Parent Interview for Externalizing Disorders in Children and Adolescents [52–54], which was conducted by telephone. Additionally, parents had to indicate the presence of either at least five out of nine symptoms of inattention, at least four out of nine symptoms of hyperactivity-impulsivity, at least eight out of 18 ADHD symptoms (inattentive or hyperactive-impulsive), or/and at least four out of eight ODD symptoms in their child. Exclusion criteria for the children were the diagnosis of a serious mental illness, the diagnosis of an autism spectrum disorder, or the need for inpatient treatment as indicated by the health care provider. The terms "parents" or "mother/father" include not only biological parents but also other primary caregivers of the child who are most likely to perform a parenting function for the child.

### Measures

The participating parents completed all questionnaires used for the current analyses online; there was no face-to-face contact with any of the participants. The parents rated their child’s ADHD and ODD symptom severity on the German Symptom Checklist for Attention-Deficit/Hyperactivity Disorder (SCL-ADHD), German: “Fremdbeurteilungsbogen für Aufmerksamkeitsdefizit-/Hyperaktivitätsstörungen [52], and on the German Symptom Checklist for Disruptive Behavior Disorders (SCL-DBD), German: “Fremdbeurteilungsbogen für Störungen des Sozialverhaltens [52]. The items of both questionnaires are based on DSM-5 and ICD-10 symptom criteria. The SCL-ADHD assesses ADHD symptoms with eighteen items. From the SCL-DBD, we only applied the eight-item ODD scale. Parents rated each item on a four-point Likert-type scale ranging from 0 (not at all) to 3 (very much/particularly severe). An overall ADHD score and an overall ODD score were computed by averaging the respective item scores. Both the SCL-ADHD and the SCL-DBD have demonstrated factorial validity and satisfactory internal consistency [52, 55–57]. In the present sample, Cronbach’s α was 0.89 for the overall ADHD score and 0.88 for the ODD score.

In addition, the parents rated the extent to which they had experienced symptoms of depression, anxiety, and stress in the preceding week using the German version of the Depression Anxiety Stress Scales [58–61]. This 42-item questionnaire consists of three 14-item scales measuring the negative emotional states of depression, anxiety, and stress, respectively. Parents rated each of the items on a four-point Likert scale ranging from 0 (did not apply to me at all) to 3 (applied to me very much or most of the time). For the current analyses, item mean scores were calculated for the total scale and for the three subscales (the analyses including the subscales are only provided in the online supplement; see below). The factor structure of the DASS has been confirmed by both exploratory and confirmatory factor analyses [59]. Moreover, the DASS subscales have demonstrated high internal consistency (α ≥ 0.81) and convergent validity [59]. In the present sample, all subscales and the total score demonstrated good to very good internal consistency (Cronbach’s α for the total score: 0.95, Cronbach’s α for the subscales: 0.83–0.90). Functional and dysfunctional parenting behaviors were assessed via self-report using the German questionnaire for positive and negative parenting behavior (German: “Fragebogen zum positiven und negativen Erziehungsverhalten”, FPNE; [62]). The questionnaire comprises 21 items assessing positive parenting behaviors (i.e., behaviors to promote beneficial parent–child interactions) and 17 items assessing negative parenting behaviors (i.e., inconsistent, impulsive, and/or rigid parenting behavior). The items originate from the Management of Children’s Behavior Scale (MCBS; [63]) and the Parent Practices Scale (PPS); [64]). Moreover, the scale comprises some newly developed items, which capture aspects of behavioral parent training (e.g., handling of family rules). Parents rated all items on a four-point Likert-type scale ranging from 1 (never) to 4 (very often/most of the time); scale scores were derived by averaging the associated item scores. Several studies have demonstrated sound psychometric properties of the MCBS, the PPS, and the FPNE itself. Psychometric analyses of the MCBS supported the internal consistency, sensitivity to change, as well as the concurrent and predictive validity of the scale [63]. The PPS has also demonstrated internal consistency and construct validity [64]. The two scales of the FPNE have demonstrated satisfactory internal consistency both in previous analyses [62] and in the present sample (positive parenting: α = 0.88; negative parenting: α = 0.71).

### Statistical Analyses

To examine whether positive and negative parenting behaviors mediate the association between parental internalizing symptoms and ADHD or ODD symptoms, respectively, we conducted mediation analyses using the SPSS macro PROCESS [24]. PROCESS employs ordinary least squares (OLS) regression to estimate the model parameters. In a simple mediation model, the independent variable (*X*) influences the dependent variable (*Y*) through a mediator variable (*M*) [25]. The total effect of *X* on *Y* (*c*) is the sum of a direct effect (*c*’) and an indirect effect (*ab*) through the mediator variable [25]. The indirect effect (*ab*) is the product of two paths: the effect of *X* on *M* (*a*), and the effect of *M* on *Y* after controlling for the effect of *X* (*b*). This product *ab* can be tested for significance [25]. The direct effect (*c*’) represents the effect of *X* on *Y* when controlling for *M* [25]. Several putative mediators that are not supposed to causally influence each other may be considered together in a parallel multiple mediator model [24]. In such a model, the specific indirect effect through one of the mediator variables (*M*_i_) is the product of the paths linking *X* and *M*_i_ and *M*_i_ and *Y* (*a*_i_*b*_i_), controlling for all other mediators in the model. The specific indirect effects add up to the total indirect effect. The total effect in this model is composed by the sum of the indirect effects and the direct effect [24].

**Fig. 1:**
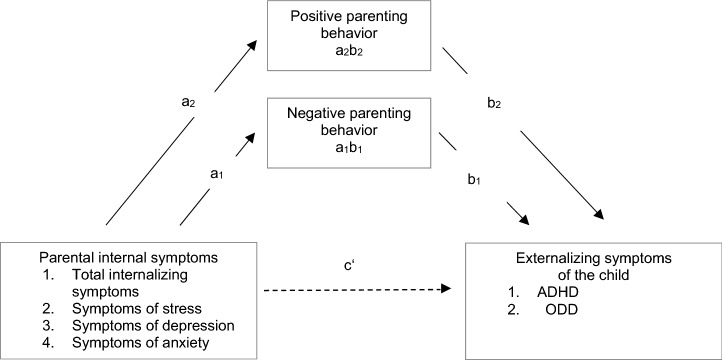
Multiple mediator model for the mediation of the association of parental internalizing symptoms and child externalizing behavior through parenting behaviors. In total, eight different models were considered: either child ADHD symptoms or child ODD symptoms were considered as dependent variable, and either total parental internalizing symptoms (symptoms of depression, anxiety, and stress combined), symptoms of depression, symptoms of anxiety, or symptoms of stress as independent variable. All models used positive parenting behavior and negative parenting behavior as parallel mediators. The results for the models including symptoms of depression, symptoms of anxiety, and symptoms of stress as independent variable are provided in the online supplement. *ADHD* attention-deficit/hyperactivity disorder, *ODD* oppositional defiant disorder

In the present study, we analyzed two separate parallel multiple mediator models, using either child ADHD symptom severity or child ODD symptom severity as the dependent variable and total parental internalizing symptoms as the independent variable. To provide an impression of the associations at the DASS subscale level, additional results for models considering either parental anxiety symptoms, depression symptoms, or stress symptoms as independent variable are presented in the online supplement. In each model, positive and negative parenting behaviors were used as parallel mediators (see Fig. 1). To interpret the indirect effects, we considered the significance of the product *ab*, but not the significance of the single paths constituting these effects, which is in line with current recommendations [24]. We report unstandardized regression coefficients and determined percentile bootstrap confidence intervals (10,000 iterations) [24]. An estimate was considered as statistically significant if the 95% confidence interval did not include zero. To enable an estimation of the size of the effects, we provide completely standardized total, direct, and indirect effects. Completely standardized effects express the unstandardized effects divided by the standard deviation of the dependent variable and multiplied by the standard deviation of the independent variable [24]. Moreover, to gain an impression of the goodness of fit of our hypothetical models, we considered the proportion of variance in the mediators explained by the independent variable and the proportion of variance in the dependent variable explained by the independent variable and the mediators taken together [24].

Usually, mediation models assume causal relationships, hypothesizing that the independent variable influences the mediator, which in turn has an effect on the dependent variable. To establish such a causal chain, it is recommended to assess the independent variable, the mediator(s), and the dependent variable in consecutive order [24, 65]. However, as we assessed all variables in our models at the same assessment point, we cannot rule out the possibility that another configuration of the models could be closer to reality. Moreover, from a theoretical perspective, it might be conceivable that parental symptoms directly affect child symptoms (e.g., due to genetic reasons), which then influence how parents behave towards the child. Therefore, we additionally examined two alternative models, in which we modeled parental internalizing symptoms as independent variable, child ADHD symptoms and child ODD symptoms as parallel mediators, and either positive parenting behaviors or negative parenting behaviors as dependent variable.

## Results

### Sample Characteristics

**Fig. 2:**
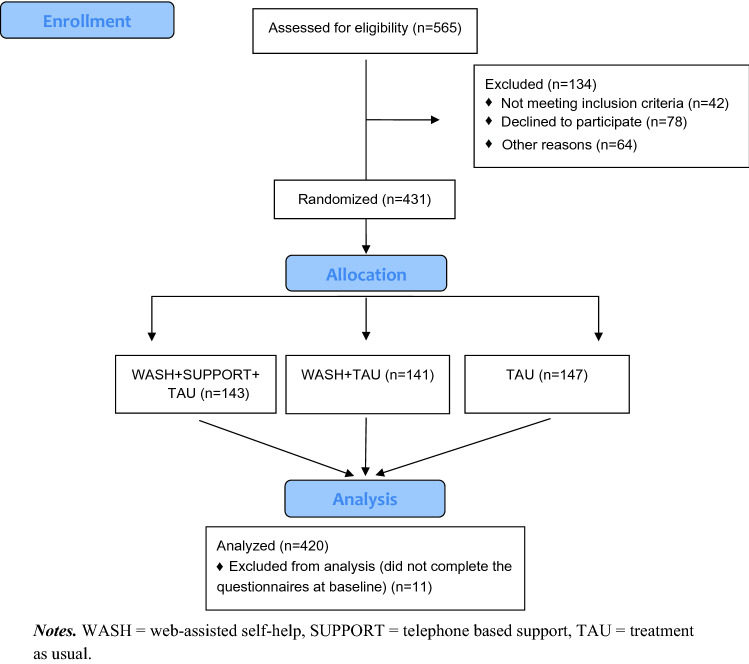
Selection of analysis sample

From January 2018 to March 2020, pediatricians and child and adolescent psychiatrists registered a total of *N* = 565 participants for the study. Of these, 431 families met the inclusion criteria, agreed to participate in the study, and were thus randomly assigned to one of the three groups. For the analyses in this article, we considered data of 420 families who subsequently completed the online questionnaires (see Fig. 2).

On average, the 420 children (81.7% male) were 9.4 years old (*SD* = 1.7). The referring physicians indicated that 58.3% of the children met the diagnostic criteria for ADHD (F90.0), 13.6% of the children had been diagnosed with a hyperkinetic conduct disorder (F90.1), 2.9% had a diagnosis of other specified behavioral and emotional disorders (F98.8), 1.2% met the diagnostic criteria for ADHD other type or unspecified type (F90.8; F90.9), and 0.5% of the children had been diagnosed with ODD (F91.3). Moreover, the referring physicians suspected a diagnosis of ADHD in 23.6% of the children. About half of the children (54.8%) were on ADHD medication and 26% of the parents indicated that their child was currently undergoing psychotherapy. In 88.3% of the cases, the participating parent was the biological mother of the child with externalizing behavior problems; 7.6% of the participants were biological fathers, 3.3% were adoptive mothers, and 0.7% were grandparents or other caregivers. The mean age of the participating parents was 41.5 years (*SD* = 5.8). Most of the children (69.5%) lived with both of their parents, 14.3% lived with their mother only, 1% lived with their father only, 11% lived with their mother and her partner, 0.5% lived with their father and his partner, 0.2% lived with their grandparents or other relatives, and 3.6% reported different constellations (e.g., weekly rotation principle, foster care).

Notably, the participating parents reported a rather low level of internalizing symptoms as well as a rather high level of positive parenting behavior (see Table S1 in the online supplement).

### Mediation Analysis

To examine whether the association between parental internalizing symptoms and child externalizing symptoms was mediated through positive and negative parenting behaviors, we first examined a model which used child ADHD symptom severity as the dependent variable, total parental internalizing symptoms as the independent variable and positive and negative parenting behaviors as parallel mediators. This model yielded a significant total effect. Parental internalizing symptoms showed a significant negative association with positive parenting behaviors and a significant positive association with negative parenting behaviors. Moreover, there was a significant positive association between positive parenting behaviors and child ADHD symptoms. The specific indirect effect of parental internalizing symptoms on child ADHD symptoms through positive parenting behaviors was significant. In other words, a higher level of parental internalizing symptoms was associated with a lower level of positive parenting behavior, which − contrary to our expectations − led to a lower level of child ADHD symptoms. The corresponding completely standardized specific indirect effect was − 0.09, meaning that children whose parents differ by one unit in their internalizing symptoms differ in their ADHD symptom severity by 0.09 standard deviations as a result of the indirect effect through positive parenting behaviors. The specific indirect effect of parental internalizing symptoms on child ADHD symptoms through negative parenting behaviors was non-significant in this model. The direct effect of parental symptoms on child ADHD symptoms remained significant after controlling for the mediators (see Table 1). In this model, parental internalizing symptoms explained 14% of the variance in negative parenting behaviors and about 5% of the variance in positive parenting behaviors. Moreover, parental internalizing symptoms and the mediators taken together explained about 12% of the variance in child ADHD symptoms.

**Table 1:** Unstandardized regression coefficients, bootstrap confidence intervals, and model information for the multiple mediator model for the mediation of the association of parental internalizing symptoms (depression, anxiety, and stress symptoms) and child externalizing behavior through parenting behaviors (*n* = 420)

	Outcome							
	ADHD				ODD			
	Coeff	Bootstrap SE	95% bootstrap CI	Completely stand. effect	Coeff	Bootstrap SE	95% bootstrap CI	Completely stand. effect
*a*_1_	0.27*	0.03	0.20; 0.33		0.27*	0.03	0.20; 0.33	
*b*_1_	0.17	0.09	− 0.01; 0.36		0.50*	0.12	0.26; 0.75	
*a*_1_*b*_1_	0.05	0.02	− 0.002; 0.10	0.04	0.13*	0.04	0.06; 0.21	0.08
*a*_2_	− 0.19*	0.04	− 0.27; − 0.11		− 0.19*	0.04	− 0.27; − 0.11	
*b*_2_	0.26*	0.07	0.11; 0.40		− 0.10	0.10	− 0.29; 0.09	
*a*_2_*b*_2_	− 0.05*	0.02	− 0.09; − 0.02	− 0.04	0.02	0.02	− 0.02; 0.06	0.01
*c′*	0.40*	0.06	0.27; 0.52	0.31	0.21*	0.08	0.04;0.37	0.12
*c*	0.40*	0.06	0.28; 0.51	0.31	0.36*	0.08	0.20;0.52	0.22

*a*_1_ parental symptoms of depression, anxiety, and stress → negative parenting behavior, *b*_1_ negative parenting behavior → outcome, *a*_1_*b*_1_ indirect effect of parental symptoms of depression, anxiety, and stress on outcome through negative parenting behavior, *a*_2_ parental symptoms of depression, anxiety, and stress → positive parenting behavior, *b*_2_ positive parenting behavior → outcome, *a*_2_*b*_2_ indirect effect of parental symptoms of depression, anxiety, and stress on outcome through positive parenting behavior, *c*′ direct effect of parental symptoms of depression, anxiety, and stress on outcome, *c* total effect of parental symptoms of depression, anxiety, and stress on outcome, *ADHD* attention-deficit/hyperactivity disorder, *ODD* oppositional defiant disorder, *Coeff*. unstandardized regression coefficient, *SE* standard error, *CI* confidence interval

*Significant coefficient (95% CI does not include zero). The standard errors and confidence intervals for the total effects were determined without the use of bootstrap samples

Second, we examined a model using child ODD symptom severity as the dependent variable, total parental internalizing symptoms as the independent variable and positive and negative parenting behaviors as parallel mediators. This model yielded both a significant total effect and a significant direct effect after controlling for the mediators. In this model, there was a significant positive association between parental internalizing symptoms and negative parenting behaviors. Moreover, we found a significant positive association between negative parenting behaviors and child ODD symptoms. The specific indirect effect of parental internalizing symptoms on child ODD symptoms through negative parenting behaviors was also significant. That is, a higher level of parental internalizing symptoms predicted a higher level of negative parenting behaviors, which in turn led to a higher level of ODD symptoms. The corresponding completely standardized specific indirect effect was 0.08. That is, children whose parents differ by one unit in their internalizing symptoms differ in their ODD symptom severity by about one tenth of a standard deviation as a result of the specific indirect effect through negative parenting behaviors. The specific indirect effect through positive parenting behaviors was non-significant. In this model, parental internalizing symptoms and the mediators taken together accounted for about 9% of the variance in ODD symptoms.

Finally, we regarded parental internalizing symptoms on the DASS subscale level. That is, we considered either parental anxiety symptoms, depression symptoms, or stress symptoms as independent variable (with child ADHD or ODD symptoms, respectively, again modeled as dependent variable and positive and negative parenting behaviors modeled as parallel mediators). The findings for the resulting models were, on an overall level, comparable to those for the models including a composite score for parental internalizing symptoms. However, when considering child ADHD symptoms as dependent variable and either parental symptoms of depression or parental symptoms of anxiety as independent variable, we found an additional significant specific indirect effect through negative parenting behaviors. Here, a higher level of parental symptoms of depression or anxiety, respectively, predicted a higher level of negative parenting behaviors, which was in turn associated with a higher level of ADHD symptoms (see Table S2 in the online supplement).

When we reversed the mediators and outcomes, i.e. when we considered child ADHD and ODD symptoms as parallel mediators and used negative or positive parenting behaviors, respectively, as outcomes, the results were as follows (also depicted in Table 2): In both the model using positive parenting behaviors and the model using negative parenting behaviors as outcome, we detected a significant total effect and a significant direct effect when controlling for the mediators. In the model using positive parenting behaviors as outcome, we detected both a significant specific indirect effect through child ADHD symptoms and a specific indirect effect through child ODD symptoms. A higher level of parental internalizing symptoms was associated with a higher level of child ADHD symptoms, which was in turn associated with more positive parenting behaviors (completely standardized effect: 0.07). On the other hand, a higher level of parental internalizing symptoms was related to a higher level of ODD symptoms, which in turn demonstrated a negative association with positive parenting behaviors (completely standardized effect: -0.04). In the model including negative parenting behaviors as outcome, only the specific indirect effect through child ODD symptoms became significant. As stated above, a higher level of parental internalizing symptoms was associated with a higher level of child ODD symptoms. Here, a higher level of ODD symptoms was in turn related to more negative parenting behaviors (completely standardized effect: 0.05). Parental internalizing symptoms accounted for about 9% of the variance in ADHD symptoms and about 5% of the variance in ODD symptoms. Moreover, parental internalizing symptoms and the mediators taken together explained about 10% of the variance in positive parenting behaviors and about 18% of the variance in negative parenting behaviors.

**Table 2:** Unstandardized regression coefficients, bootstrap confidence intervals, and model information for the multiple mediator model for the mediation of the association of parental internalizing symptoms (depression, anxiety and stress symptoms) and parenting behavior through child externalizing behaviors (*n* = 420)

	Outcome							
	Positive parenting				Negative parenting			
	Coeff	Bootstrap SE	95% bootstrap CI	Completely stand. effect	Coeff	Bootstrap SE	95% bootstrap CI	Completely stand. effect
*a*_1_	0.40*	0.06	0.28; 0.51		0.40*	0.06	0.28; 0.51	
*b*_1_	0.15*	0.04	0.08; 0.22		− 0.02	0.03	− 0.08; 0.03	
*a*_1_*b*_1_	0.06*	0.02	0.03; 0.09	0.07	− 0.01	0.01	− 0.03; 0.01	− 0.01
*a*_2_	0.36*	0.08	0.21; 0.52		0.36*	0.08	0.21; 0.52	
*b*_2_	− 0.10*	0.03	− 0.15; − 0.04		0.09*	0.02	0.05; 0.14	
*a*_2_*b*_2_	− 0.04*	0.01	− 0.06; − 0.01	− 0.04	0.03*	0.01	0.02; 0.06	0.05
*c*′	− 0.21*	0.05	− 0.30; − 0.12	− 0.24	0.24*	0.04	0.17; 0.31	0.34
*c*	− 0.19*	0.04	− 0.27; − 0.11	− 0.22	0.27*	0.03	0.20; 0.33	0.37

*a*_1_ parental symptoms of depression, anxiety, and stress → ADHD symptoms of the child, *b*_1_ ADHD symptoms of the child → outcome, *a*_1_*b*_1_ indirect effect of parental symptoms of depression, anxiety, and stress on outcome through ADHD symptoms of the child, *a*_2_ parental symptoms of depression, anxiety, and stress → ODD symptoms of the child, *b*_2_ ODD symptoms of the child → outcome, *a*_2_*b*_2_ indirect effect of parental symptoms of depression, anxiety, and stress on outcome through ODD symptoms of the child, *c*′ direct effect of parental symptoms of depression, anxiety, and stress on outcome, *c* total effect of parental symptoms of depression, anxiety, and stress on outcome, *ADHD* attention-deficit/hyperactivity disorder, *ODD* oppositional defiant disorder, *Coeff*. unstandardized regression coefficient, *SE* standard error, *CI* confidence interval

*Significant coefficient (95% CI does not include zero). The standard errors and confidence intervals for the total effects were determined without the use of bootstrap samples

## Discussion

The present study aimed to illuminate the mechanisms underlying the often-found association between parental internalizing symptoms and child externalizing symptoms by analyzing the mediation of this association by positive and negative parenting behaviors in a clinical sample of school-age children with elevated levels of externalizing behavior problems. The analyses revealed significant associations between parental internalizing symptoms and both child ADHD and child ODD symptoms. However, differential mediation effects were detected for the different outcome variables. While the relationship between parental internalizing symptoms (depression, anxiety, and stress) and child ADHD symptoms was mediated by positive parenting behaviors (small, negative indirect effect), a small positive indirect effect of parental internalizing symptoms on child ODD symptoms through negative parenting behaviors was detected. The indirect effects of the global measures of parental internalizing symptoms on child ADHD symptoms through negative parenting behaviors and on ODD symptoms through positive parenting behaviors were non-significant. However, when considering parental internalizing symptoms on the subscale level, we additionally detected a significant effect of both parental symptoms of depression and parental symptoms of anxiety on child ADHD symptoms through negative parenting behavior.

To sum up, in line with our expectations, our results particularly underline the role of negative parenting behavior in mediating the association between parental internalizing symptoms and child ODD symptoms, respectively. Higher levels of parental internalizing symptoms (only symptoms of depression and anxiety in the model using ADHD as outcome) were associated with a higher level of negative parenting behavior, which was in turn associated with more severe child ADHD or ODD symptoms, respectively. This finding is consistent with the results of some previous mediation studies [41, 42] and with one of the mediating mechanisms proposed by Goodman and Gotlib [22, 23], namely the mediation of the impact of parental symptoms on child symptoms by parental behavior. However, the mediation effects found in the present study were rather small. Furthermore, in all models, parental internalizing symptoms and the mediators taken together explained only a small amount of the variance in child ADHD or ODD symptoms, respectively, indicating a rather poor data fit of the proposed models. As such, there might be other (additional) variables accounting for the association between parental internalizing and child externalizing symptoms, for example a common genetic disposition and/or environmental factors.

On a descriptive level, the mediation effects through negative parenting behavior were somewhat larger in the models using ODD symptoms as outcome compared to the models using ADHD symptoms as outcome, and the mediation effect in the ADHD model was non-significant when considering a global score of parental internalizing symptoms as predictor (which additionally comprised parental symptoms of stress). This might be explained by the assumption that ADHD symptoms are more strongly determined by biological or genetic factors [45, 46].

Regarding the mediation of the association between parental symptoms and child ADHD symptoms by positive parenting behavior, we found that with increasing parental internalizing symptoms, positive parenting behavior was reduced (negative correlation). Contrary to our expectations, however, positive parenting behavior was positively associated with ADHD symptoms. In other words, with more pronounced positive parenting behavior, more severe ADHD symptoms were observed in the child. This second path contradicts the results of previous studies, which reported that lower levels of aspects of positive parenting behavior were associated with higher ADHD symptom severity [31, 34].

The cross-sectional nature of our data complicates the justification of the causal sequence proposed in the mediation models; we cannot rule out that another sequence of the mediators and outcomes might be closer to reality. The examination of alternative model configurations using parental internalizing symptoms as independent variable, child ADHD and ODD symptoms as parallel mediators, and either positive parenting behavior or negative parenting behavior as outcome yielded significant specific indirect effects through both ADHD and ODD symptoms in the model including positive parenting behavior and a significant specific indirect effect through ODD symptoms in the model including negative parenting behavior. While the direction of the indirect effect through ADHD is hard to interpret (more severe ADHD symptoms were associated with a higher level of positive parenting behavior), the indirect effects through child ODD symptoms might make sense from a theoretical point of view. It is conceivable that parental internalizing symptoms lead to child externalizing symptoms, e.g., as they share a common genetic basis, and that child externalizing symptoms, in turn, affect the way parents behave towards the child [66]. Future studies should use longitudinal data to further clarify the relation and sequence of the variables used in the models in this study, and also consider the possibility of reciprocal associations.

This study has several limitations. First, the major limitation is the cross-sectional nature of the data, which does not allow for causal interpretations. As we found some reasonably interpretable results in both our original analyses and the analyses with reversed mediators and outcomes, future studies using longitudinal data are required. Such studies would have the potential to illuminate the possibly reciprocal and complex associations between internalizing symptoms of the parents, parenting behavior, and externalizing symptoms of the child. Preferably, these studies should concentrate on children at risk of developing externalizing behavior and begin treatment before the symptoms manifest.

Second, the results are limited by the fact that all questionnaires were completed by the parents, thus reflecting parental judgment only, which might be prone to bias by socially desirable responding or dissimulation tendencies. One previous study compared self-judgment to observed judgment, and only found a significant correlation for parental warmth. Observations of parental control practices, which include inconsistency, were not significantly associated with self-judgment of these behaviors [67]. Another study showed no correlation between parent and child judgment of parenting behavior [68]. Moreover, a previous study failed to find significant effects for the mediation of the association between depressive symptoms of the parents and child internalizing and externalizing symptoms through observed parent–child interactions [43]. However, the latter study had several shortcomings, including possibly insufficient power to detect a mediating effect [43]. Taken together, the results of the latter study and the present study highlight the need to consider different sources of information (e.g., clinical ratings and observations of parental behavior) in larger clinical samples in future studies. Moreover, as the parents in the present study scored very high on the positive parenting behavior scale, their ratings may be subject to a ceiling effect [69].

Third, positive and negative parenting behavior were assessed on a fairly global level in the current study. Future studies might benefit from a more differentiated assessment of parenting behavior. For example, a questionnaire similar to the Parenting Styles and Dimensions Questionnaire (PSDQ) [70], but specifically related to externalizing disorders, may be beneficial, as these children pose special challenges to parenting. Besides the format (observations or clinical ratings), a wider range of categories (including some content from the FPNE as well) could be created: for example, abilities of the parents to “bond and respond”, consistency, dealing with boundaries and rules, dispute culture and possibilities for autonomy.

Fourth, our study did not consider potential moderators of the mediation effects. For example, parental externalizing psychopathology might affect the mediation process. A previous study found that women with ADHD symptoms have significantly more difficulties in raising their children than women without ADHD [71]. Unfortunately, ADHD symptoms of the parents were not recorded within the present study.

Fifth, another limitation is the underrepresentation of fathers in the sample. Although we imposed no restrictions concerning the gender of the parent participating in our study, the sample mostly comprised mothers. Therefore, our findings cannot be easily transferred to associations of fathers’ psychopathology and parenting behavior with the child’s symptoms. Similarly, no conclusions can be drawn concerning the effects on girls (comprising only 18.3% in the present sample) with ODD/ADHD.

## Summary

Numerous studies have demonstrated associations between parents’ internalizing symptoms and their children’s ADHD and ODD symptoms. Moreover, low levels of positive parenting behavior and high levels of negative parenting behavior have been linked to child externalizing symptoms. The aim of this study was to analyze whether the associations between parental internalizing symptoms (depression, anxiety, stress) and child symptoms of ADHD or ODD are mediated by positive and negative parenting behaviors. Cross-sectional data of 420 parents of children (age 6–12 years) with elevated levels of externalizing symptoms were collected within a randomized controlled trial. Measures included parent ratings of their internalizing symptoms and parenting behaviors and of their child’s externalizing symptoms. Two mediation models were examined, one including ADHD symptoms and one including ODD symptoms as dependent variable. Parental internalizing symptoms were modeled as the independent variable, and positive and negative parenting behaviors were modeled as parallel mediators. Regression analyses yielded a significant indirect effect of parental internalizing symptoms on child ODD symptoms through negative parenting behavior and a significant indirect effect on ADHD symptoms through positive parenting behavior. However, the direction of the latter effect was contrary to our expectations (i.e., it encompassed a positive association between positive parenting and ADHD symptom severity). Thus, this study mainly supports the assumption of negative parenting behavior as a mediator of the association between parental symptoms and child ODD symptoms. The main limitation of the study pertains to the cross-sectional nature of the analyses. Future studies should use prospective designs and consider reciprocal associations.

### Supplementary Information

Below is the link to the electronic supplementary material.

Supplementary file1 (DOCX 23 KB)

## References

[1] Polanczyk GV, Salum GA, Sugaya LS (2015). Annual research review: a meta-analysis of the worldwide prevalence of mental disorders in children and adolescents. J Child Psychol Psychiatry.

[2] American Psychiatric Association (2013) Diagnostic and statistical manual of mental disorders. American Psychiatric Association

[3] World Health Organization (2015) International statistical classification of diseases and related health problems, 10th revision, 5th edn, 2016. World Health Organization

[4] Riley M, Ahmed S, Locke A (2016). Common questions about oppositional defiant disorder. Am Fam Physician.

[5] Gillberg C, Gillberg IC, Rasmussen P (2004). Co-existing disorders in ADHD—implications for diagnosis and intervention. Eur Child Adolesc Psychiatry.

[6] Chronis AM, Lahey BB, Pelham WE (2007). Maternal depression and early positive parenting predict future conduct problems in young children with attention-deficit/hyperactivity disorder. Dev Psychol.

[7] Cheung K, Theule J (2016). Parental psychopathology in families of children with ADHD: a meta-analysis. J Child Fam Stud.

[8] Deault LC (2010). A systematic review of parenting in relation to the development of comorbidities and functional impairments in children with attention-deficit/hyperactivity disorder (ADHD). Child Psychiatry Hum Dev.

[9] Theule J, Wiener J, Tannock R (2013). Parenting stress in families of children with ADHD. J Emot Behav Disord.

[10] Wüstner A, Otto C, Schlack R (2019). Risk and protective factors for the development of ADHD symptoms in children and adolescents: results of the longitudinal BELLA study. PLoS ONE.

[11] Cussen A, Sciberras E, Ukoumunne OC (2012). Relationship between symptoms of attention-deficit/hyperactivity disorder and family functioning: a community-based study. Eur J Pediatr.

[12] Civic D, Holt VL (2000). Maternal depressive symptoms and child behavior problems in a nationally representative normal birthweight sample. Matern Child Health J.

[13] Elgar FJ, Curtis LJ, McGrath PJ (2003). Antecedent-consequence conditions in maternal mood and child adjustment: a four-year cross-lagged study. J Clin Child Adolesc Psychol.

[14] Harvey EA, Metcalfe LA, Herbert SD (2011). The role of family experiences and ADHD in the early development of oppositional defiant disorder. J Consult Clin Psychol.

[15] Galéra C, Côté SM, Bouvard MP (2011). Early risk factors for hyperactivity-impulsivity and inattention trajectories from age 17 months to 8 years. Arch Gen Psychiatry.

[16] Kouros CD, Garber J (2010). Dynamic associations between maternal depressive symptoms and adolescents' depressive and externalizing symptoms. J Abnorm Child Psychol.

[17] Podolski CL, Nigg JT (2001). Parent stress and coping in relation to child ADHD severity and associated child disruptive behavior problems. J Clin Child Psychol.

[18] Williford AP, Calkins SD, Keane SP (2007). Predicting change in parenting stress across early childhood: child and maternal factors. J Abnorm Child Psychol.

[19] Antúnez Z, La Osa N, de, Granero R (2018). Reciprocity between parental psychopathology and oppositional symptoms from preschool to middle childhood. J Clin Psychol.

[20] Gross HE, Shaw DS, Burwell RA (2009). Transactional processes in child disruptive behavior and maternal depression: a longitudinal study from early childhood to adolescence. Dev Psychopathol.

[21] Mackler JS, Kelleher RT, Shanahan L (2015). Parenting stress, parental reactions, and externalizing behavior from ages 4 to 10. J Marriage Fam.

[22] Goodman SH, Gotlib IH (1999). Risk for psychopathology in the children of depressed mothers: a developmental model for understanding mechanisms of transmission. Psychol Rev.

[23] Goodman SH, Rouse MH, Connell AM (2011). Maternal depression and child psychopathology: a meta-analytic review. Clin Child Fam Psychol Rev.

[24] Hayes AF (2018) Introduction to mediation, moderation, and conditional process analysis: a regression-based approach, Second edition. Methodology in the social sciences. Guilford Press, New York

[25] Preacher KJ, Hayes AF (2008). Asymptotic and resampling strategies for assessing and comparing indirect effects in multiple mediator models. Behav Res Methods.

[26] Patterson GR, DeBaryshe BD, Ramsey E (1989). A developmental perspective on antisocial behavior. Am Psychol.

[27] Buschgens CJM, van Aken MAG, Swinkels SHN (2010). Externalizing behaviors in preadolescents: familial risk to externalizing behaviors and perceived parenting styles. Eur Child Adolesc Psychiatry.

[28] Gaik LP, Abdullah MC, Elias H (2010). Development of antisocial behaviour. Procedia Soc Behav Sci.

[29] Burt SA, Clark DA, Gershoff ET (2021). Twin differences in harsh parenting predict youth's antisocial behavior. Psychol Sci.

[30] Pfiffner LJ, McBurnett K, Rathouz PJ (2005). Family correlates of oppositional and conduct disorders in children with attention deficit/hyperactivity disorder. J Abnorm Child Psychol.

[31] Hawes DJ, Dadds MR, Frost ADJ (2013). Parenting practices and prospective levels of hyperactivity/inattention across early- and middle-childhood. J Psychopathol Behav Assess.

[32] Breaux RP, Harvey EA (2019). A longitudinal study of the relation between family functioning and preschool ADHD symptoms. J Clin Child Adolesc Psychol.

[33] Kim DH, Yoo IY (2013). Relationship between attention deficit hyperactive disorder symptoms and perceived parenting practices of school-age children. J Clin Nurs.

[34] Dvorsky MR, Langberg JM (2016). A review of factors that promote resilience in youth with ADHD and ADHD symptoms. Clin Child Fam Psychol Rev.

[35] Reuben JD, Shaw DS, Neiderhiser JM (2016). Warm parenting and effortful control in toddlerhood: independent and interactive predictors of school-age externalizing behavior. J Abnorm Child Psychol.

[36] Letourneau N, Salmani M, Duffett-Leger L (2010). Maternal depressive symptoms and parenting of children from birth to 12 years. West J Nurs Res.

[37] Waylen A, Stewart-Brown S (2010). Factors influencing parenting in early childhood: a prospective longitudinal study focusing on change. Child Care Health Dev.

[38] Cornish AM, McMahon CA, Ungerer JA (2006). Maternal depression and the experience of parenting in the second postnatal year. J Reprod Infant Psychol.

[39] Kashdan TB, Jacob RG, Pelham WE (2004). Depression and anxiety in parents of children with ADHD and varying levels of oppositional defiant behaviors: modeling relationships with family functioning. J Clin Child Adolesc Psychol.

[40] Trepat E, Granero R, Ezpeleta L (2014). Parenting practices as mediating variables between parents' psychopathology and oppositional defiant disorder in preschoolers. Psicothema.

[41] Elgar FJ, Mills RSL, McGrath PJ (2007). Maternal and paternal depressive symptoms and child maladjustment: the mediating role of parental behavior. J Abnorm Child Psychol.

[42] Dette-Hagenmeyer DE, Reichle B (2014). Parents' depressive symptoms and children's adjustment over time are mediated by parenting, but differentially for fathers and mothers. Eur J Dev Psychol.

[43] van Doorn MMEM, Kuijpers RCWM, Lichtwarck-Aschoff A (2016). Does mother–child interaction mediate the relation between maternal depressive symptoms and children's mental health problems?. J Child Fam Stud.

[44] Forehand R, Lafko N, Parent J (2014). Is parenting the mediator of change in behavioral parent training for externalizing problems of youth?. Clin Psychol Rev.

[45] Barkley RA (2006). Attention-deficit hyperactivity disorder: a handbook for diagnosis and treatment.

[46] de Zeeuw EL, van Beijsterveldt CEM, Lubke GH (2015). Childhood ODD and ADHD behavior: the effect of classroom sharing, gender, teacher gender and their interactions. Behav Genet.

[47] Harvey EA, Breaux RP, Lugo-Candelas CI (2016). Early development of comorbidity between symptoms of attention-deficit/hyperactivity disorder (ADHD) and oppositional defiant disorder (ODD). J Abnorm Psychol.

[48] Faraone SV, Larsson H (2019). Genetics of attention deficit hyperactivity disorder. Mol Psychiatry.

[49] Sprich S, Biederman J, Crawford MH (2000). Adoptive and biological families of children and adolescents with ADHD. J Am Acad Child Adolesc Psychiatry.

[50] Knopik VS, Bidwell LC, Flessner C (2014). DSM-IV defined conduct disorder and oppositional defiant disorder: an investigation of shared liability in female twins. Psychol Med.

[51] Döpfner M, Wähnke L, Klemp M-T (2020). Efficacy of web-assisted self-help for parents of children with ADHD (WASH) - a three-arm randomized trial under field/routine care conditions in Germany. BMC Psychiatry.

[52] Döpfner M, Görtz-Dorten A (2017) DISYPS-III: Diagnostik-System für psychische Störungen nach ICD-10 und DSM-5 für Kinder und Jugendliche - III : Manual. Hogrefe, Bern

[53] Thöne A-K, Görtz-Dorten A, Altenberger P (2020). Toward a dimensional assessment of externalizing disorders in children: reliability and validity of a semi-structured parent interview. Front Psychol.

[54] Görtz-Dorten A, Döpfner M, Thöne A-K (2021) DISYPS-ILF: Interview-Leitfäden zum Diagnostik-System für psychische Störungen nach DSM-5 für Kinder- und Jugendliche. Hogrefe, Bern

[55] Ravens-Sieberer U, Wille N, Erhart M (2008). Prevalence of mental health problems among children and adolescents in Germany: results of the BELLA study within the National Health Interview and Examination Survey. Eur Child Adolesc Psychiatry.

[56] Görtz-Dorten A, Ise E, Hautmann C (2014). Psychometric properties of a German parent rating scale for oppositional defiant and conduct disorder (FBB-SSV) in clinical and community samples. Child Psychiatry Hum Dev.

[57] Erhart M, Döpfner M, Ravens-Sieberer U (2008). Psychometric properties of two ADHD questionnaires: comparing the Conners' scale and the FBB-HKS in the general population of German children and adolescents–results of the BELLA study. Eur Child Adolesc Psychiatry.

[58] Nilges P, Essau C (2015). Die Depressions-Angst-Stress-Skalen: Der DASS–ein Screeningverfahren nicht nur für Schmerzpatienten (Depression, anxiety and stress scales: DASS–A screening procedure not only for pain patients). Schmerz.

[59] Lovibond PF, Lovibond SH (1995). The structure of negative emotional states: comparison of the Depression Anxiety Stress Scales (DASS) with the Beck Depression and Anxiety Inventories. Behav Res Ther.

[60] Lovibond SH, Lovibond PF (1995) Manual for the Depression Anxiety Stress Scales (DASS), 2nd edn. Psychology foundation monograph. Psychology Foundation of Australia, Sydney

[61] Köppe EC (2001) Glückliche Eltern - liebe Kinder? Braunschweig, Techn. Univ., Diss., 2001

[62] Imort S, Hautmann C, Greimel L et al (2014) Der Fragebogen zum positiven und negativen Erziehungsverhalten (FPNE): Eine psychometrische Zwischenanalyse.: Poster zum 32 Symposium der Fachgruppe Klinische Psychologie und Psychotherapie der Deutschen Gesellschaft für Psychologie, Braunschweig

[63] Perepletchikova F, Kazdin AE (2004). Assessment of parenting practices related to conduct problems: development and validation of the management of children's behavior scale. J Child Fam Stud.

[64] Strayhorn JM, Weidman CS (1988). A Parent Practices Scale and its relation to parent and child mental health. J Am Acad Child Adolesc Psychiatry.

[65] Zhang Z, Zheng C, Kim C et al (2016) Causal mediation analysis in the context of clinical research. Ann Transl Med 4:425. 10.21037/atm.2016.11.11 10.21037/atm.2016.11.11 PMC5124624 27942516

[66] Mikami AY, Jack A, Emeh CC (2010). Parental influence on children with attention-deficit/hyperactivity disorder: I. Relationships between parent behaviors and child peer status. J Abnorm Child Psychol.

[67] Arney FM (2004) A comparison of direct observation and self-report measures of parenting behaviour

[68] Kwon M (2009). Comparison of child-rearing attitudes of parents and problem behavior of children as perceived by parents and children. J Korean Acad Child Health Nurs.

[69] Döring N, Bortz J, Pöschl S (2016) Forschungsmethoden und Evaluation in den Sozial- und Humanwissenschaften, 5., vollst. überarb., aktualisierte und erw. Aufl. SpringerLink Bücher. Springer, Berlin [u.a.]

[70] Robinson C, Mandleco B, Roper S (2001). The Parenting Styles and Dimensions Questionnaire (PSDQ). Handb Fam Meas Tech.

[71] Banks T, Ninowski JE, Mash EJ (2008). Parenting behavior and cognitions in a community sample of mothers with and without symptoms of attention-deficit/hyperactivity disorder. J Child Fam Stud.

