# Supplementary material for: Parenting Behaviors as Mediators of the Association Between Parental Internalizing Symptoms and Child Externalizing Symptoms
Source: Child Psychiatry Hum Dev. 2022 Oct 28;55(4):916–28. doi: 10.1007/s10578-022-01462-0 (PMC11245424; doi:10.1007/s10578-022-01462-0)
Supplement: Supplementary file 1 — Supplementary file1 (DOCX 23 KB) [file 10578_2022_1462_MOESM1_ESM.docx]

**Supporting Information for *Parenting Behaviors as Mediators of the Association between Parental Internalizing Symptoms and Child Externalizing Symptoms*** by Klemp et al. 2022

**Table S1**

Descriptive statistics for child externalizing behavior problems, parenting behavior and internal parental psychopathology (depression, anxiety and stress)

|  |  | *M* | *SD* | Range (theoretical) |
| --- | --- | --- | --- | --- |
| Symptom Checklists for ADHD and DBD (SCL-ADHD, SCL-DBD) | ADHD | 1.76 | 0.52 | 0.4 - 3.0 (0-3) |
|  | ODD | 1.50 | 0.68 | 0.0 - 3.0 (0-3) |
| Depression Anxiety Stress Scales (DASS) | Stress | 0.79 | 0.47 | 0.0 - 2.5 (0-3) |
|  | Anxiety | 0.51 | 0.40 | 0.0 - 2.1 (0-3) |
|  | Depression | 0.56 | 0.40 | 0.0 - 2.4 (0-3) |
|  | Total | 0.62 | 0.41 | 0.0 - 2.3 (0-3) |
| Questionnaire for Positive and Negative Parenting Behavior (FPNE) | Positive parenting behavior | 3.02 | 0.35 | 1.9 - 3.9 (1-4) |
|  | Negative parenting behavior | 1.93 | 0.29 | 1.2 - 2.8 (1-4) |

Note. Sample size n = 420; ADHD = attention-deficit/hyperactivity disorder, ODD = oppositional defiant disorder.

**Table S2**

Multiple mediator models for the mediation of the impact of parental symptoms of depression, anxiety and stress on child externalizing symptoms through parenting behaviors

|  |  | Dependent variable | | | | | | | |
| --- | --- | --- | --- | --- | --- | --- | --- | --- | --- |
|  |  | ADHD | | | | ODD | | | |
| Independent variable |  | Coeff. | Bootstrap SE | 95%  Bootstrap CI | Completely  stand. effect | Coeff. | Bootstrap SE | 95%  Bootstrap CI | Completely  stand. effect |
| Parental symptoms  of depression | *a*_1_ | 0.22* | 0.03 | 0.15; 0.29 |  | 0.22* | 0.03 | 0.15; 0.29 |  |
|  | *b*_1_ | 0.22* | 0.09 | 0.04; 0.40 |  | 0.52* | 0.13 | 0.27; 0.76 |  |
|  | *a*_1_*b*_1_ | 0.05* | 0.02 | 0.01; 0.09 | 0.04 | 0.12* | 0.03 | 0.06; 0.19 | 0.07 |
|  | *a*_2_ | -0.15* | 0.05 | -0.24; -0.06 |  | -0.15* | 0.05 | -0.24; -0.06 |  |
|  | *b*_2_ | 0.24* | 0.08 | 0.09; 0.39 |  | -0.10 | 0.10 | -0.30; 0.10 |  |
|  | *a*_2_*b*_2_ | -0.04* | 0.02 | -0.07; -0.01 | -0.03 | 0.02 | 0.02 | -0.02; 0.05 | 0.01 |
|  | *c*‘ | 0.35* | 0.06 | 0.23; 0.47 | 0.27 | 0.20* | 0.08 | 0.04; 0.35 | 0.12 |
|  | *c* | 0.37* | 0.06 | 0.25; 0.48 | 0.28 | 0.33* | 0.08 | 0.17; 0.49 | 0.20 |
| Parental symptoms  of anxiety | *a*_1_ | 0.25* | 0.04 | 0.18; 0.32 |  | 0.25* | 0.04 | 0.18; 0.32 |  |
|  | *b*_1_ | 0.20* | 0.09 | 0.03; 0.38 |  | 0.51* | 0.13 | 0.26; 0.76 |  |
|  | *a*_1_*b*_1_ | 0.05* | 0.02 | 0.01; 0.10 | 0.04 | 0.13* | 0.04 | 0.06; 0.20 | 0.07 |
|  | *a*_2_ | -0.18* | 0.05 | -0.27; -0.09 |  | -0.18* | 0.05 | -0.27; -0.09 |  |
|  | *b*_2_ | 0.26* | 0.07 | 0.11; 0.40 |  | -0.10 | 0.10 | -0.30; 0.11 |  |
|  | *a*_2_*b*_2_ | -0.05* | 0.02 | -0.09; -0.02 | -0.03 | 0.02 | 0.02 | -0.02; 0.06 | 0.01 |
|  | *c*‘ | 0.38* | 0.06 | 0.27; 0.50 | 0.29 | 0.21* | 0.08 | 0.05; 0.37 | 0.12 |
|  | *c* | 0.38* | 0.06 | 0.26; 0.50 | 0.29 | 0.35* | 0.08 | 0.19; 0.51 | 0.21 |
| Parental symptoms  of stress | *a*_1_ | 0.25* | 0.03 | 0.20; 0.31 |  | 0.25* | 0.03 | 0.20; 0.31 |  |
|  | *b*_1_ | 0.15 | 0.09 | -0.03; 0.34 |  | 0.50* | 0.13 | 0.24; 0.76 |  |
|  | *a*_1_*b*_1_ | 0.04 | 0.02 | -0.01; 0.09 | 0.04 | 0.13* | 0.04 | 0.06; 0.20 | 0.09 |
|  | *a*_2_ | -0.18* | 0.04 | -0.25; -0.11 |  | -0.18* | 0.04 | -0.25; -0.11 |  |
|  | *b*_2_ | 0.26* | 0.07 | 0.12; 0.41 |  | -0.10 | 0.10 | -0.29; 0.11 |  |
|  | *a*_2_*b*_2_ | -0.05* | 0.02 | -0.08; -0.02 | -0.04 | 0.02 | 0.02 | -0.02; 0.06 | 0.01 |
|  | *c*‘ | 0.34* | 0.06 | 0.23; 0.45 | 0.31 | 0.16* | 0.07 | 0.02; 0.30 | 0.11 |
|  | *c* | 0.33* | 0.05 | 0.23; 0.44 | 0.30 | 0.31* | 0.07 | 0.17; 0.44 | 0.21 |

***Note.*** Sample size *n* = 420*.* *a*_1_: independent variable 🡪 negative parenting behavior, *b*_1_: negative parenting behavior 🡪 dependent variable, *a*_1_*b*_1_: indirect effect of independent variable on dependent variable through negative parenting behavior, *a*_2_: independent variable 🡪 positive parenting behavior, *b*_2_: positive parenting behavior 🡪 dependent variable, *a*_2_*b*_2_: indirect effect of independent variable on dependent variable through positive parenting behavior, *c*‘: direct effect of independent variable on dependent variable, *c*: total effect of independent variable on dependent variable, ADHD = attention-deficit/hyperactivity disorder, ODD = oppositional defiant disorder, Coeff. = unstandardized regression coefficient, SE = standard error, CI = confidence interval. * significant coefficient (95% CI). The standard errors and confidence intervals for the total effects were determined without the use of bootstrap samples.
